# Neural coding in the visual system of *Drosophila melanogaster*: How do small neural populations support visually guided behaviours?

**DOI:** 10.1371/journal.pcbi.1005735

**Published:** 2017-10-10

**Authors:** Alex D. M. Dewar, Antoine Wystrach, Andrew Philippides, Paul Graham

**Affiliations:** 1 Department of Informatics, University of Sussex, Falmer, Brighton, United Kingdom; 2 Centre de Recherches sur la Cognition Animale, Centre National de la Recherche Scientifique, Université Paul Sabatier, Toulouse, France; 3 School of Life Sciences, University of Sussex, Falmer, Brighton, United Kingdom; University College London, UNITED KINGDOM

## Abstract

All organisms wishing to survive and reproduce must be able to respond adaptively to a complex, changing world. Yet the computational power available is constrained by biology and evolution, favouring mechanisms that are parsimonious yet robust. Here we investigate the information carried in small populations of visually responsive neurons in *Drosophila melanogaster*. These so-called ‘ring neurons’, projecting to the ellipsoid body of the central complex, are reported to be necessary for complex visual tasks such as pattern recognition and visual navigation. Recently the receptive fields of these neurons have been mapped, allowing us to investigate how well they can support such behaviours. For instance, in a simulation of classic pattern discrimination experiments, we show that the pattern of output from the ring neurons matches observed fly behaviour. However, performance of the neurons (as with flies) is not perfect and can be easily improved with the addition of extra neurons, suggesting the neurons’ receptive fields are not optimised for recognising abstract shapes, a conclusion which casts doubt on cognitive explanations of fly behaviour in pattern recognition assays. Using artificial neural networks, we then assess how easy it is to decode more general information about stimulus shape from the ring neuron population codes. We show that these neurons are well suited for encoding information about size, position and orientation, which are more relevant behavioural parameters for a fly than abstract pattern properties. This leads us to suggest that in order to understand the properties of neural systems, one must consider how perceptual circuits put information at the service of behaviour.

## Author summary

A general problem in neuroscience is understanding how sensory systems organise information to be at the service of behaviour. Computational approaches can be useful for such studies as they allow one to simulate the sensory experience of a behaving animal whilst considering how sensory information should be encoded. In flies, small subpopulations of identifiable neurons are known to be necessary for particular visual tasks, and the response properties of these populations have now been described in detail. Surprisingly, these populations are small, with only 14 or 28 neurons each, which suggests something of a sensory bottleneck. In this paper, we consider how the population code from these neurons relates to the information required to control specific behaviours. We conclude that, despite previous claims, flies are unlikely to possess a general-purpose pattern-learning ability. However, implicit information about the shape and size of objects, which is necessary for many ecologically important visually guided behaviours, does pass through the sensory bottleneck. These findings show that nervous systems can be particularly economical when specific populations of cells are paired with specific visual behaviours. This is a general-interest finding for computer vision and biomimetics, as well as sensory neuroscience.

## Introduction

As with many animals, vision plays a key role in a number of behaviours performed by the fruit fly *Drosophila melanogaster*, including mate-recognition [1], place homing [2], visual course control [3], collision-avoidance [4], landing [4] and escaping a looming object (like a rolled newspaper, for example) [5]. The benefit of studying these visually guided behaviours in *Drosophila* is the range of neurogenetic techniques which give a realistic chance of understanding the neural circuits that underpin them. With that goal in mind, we focus on work by Seelig and Jayaraman [6] which mapped the receptive fields (RFs) of a set of visually responsive neurons: the ring neurons of the ellipsoid body. These neurons are necessary and sufficient for a range of complex behaviours, including short term spatial memory, pattern discrimination and place memory [2, 7–9], and yet are surprisingly small in number. To understand their role in these behaviours, we used modelling to bridge the gap between neurogenetic data and behaviour by evaluating ring neuron responses during simulations of fly experiments. In this way we investigate how small populations of visual neurons in *Drosophila*, which might represent a sensory bottleneck, can still provide behaviourally relevant information.

In laboratory assays, flies show interesting spontaneous visual behaviours. For instance, flies orient towards bar stimuli [10, 11] and in a circular arena with two diametrically opposed bars will walk between them until exhaustion [12]. The attraction to vertical bars decreases as the bar is shortened and flies are strongly repulsed by small spots [13]. In addition, a number of studies have investigated the process of pattern recognition and its neural underpinnings [7, 14, 15]. Flies seem to possess a form of pattern memory analogous to the better-studied pattern memory of bees [16–18]. Interestingly, both bees [19] and flies [14] systematically fail to discriminate certain pattern pairs.

These visual behaviours require the central complex, a major neuropil which comprises the ellipsoid body, the fan-shaped body, the paired noduli and the protocerebral bridge [20]. The central complex is thought to be involved primarily in spatial representation, action selection and mediation between visual input and motor output [21]. One class of neurons with projections in the ellipsoid body is the ‘ring neurons’, which are known to be involved in certain visual behaviours (R1: place homing [2, 22, 23]; R2/R4m: pattern recognition [7, 14, 15]; R3/R4: bar direction memory [8]). Here we investigate how the ring neurons might contribute to behaviour, by simulating the visual input as it would be processed by this small population of visually responsive cells. In particular, we can address why flies are unable to discriminate certain pattern pairs, whether these subpopulations of neurons are optimised for pattern recognition and, if not, what visually guided behaviours these cells are suited to.

In order to do this, we leverage research which has described the RF properties of two classes of ring neuron in the *Drosophila* ellipsoid body [6]. The two subtypes of neuron investigated were the R2 and the R4d ring neurons, of which only 28 and 14, respectively, were responsive to visual stimuli. The cells were found to possess RFs that were large, centred in the ipsilateral portion of the visual field and with forms similar to those of mammalian simple cells [24] (for details of how the RFs were estimated, see Materials and methods). Like simple cells, many of these neurons showed strong orientation tuning and some were sensitive to the direction of motion of stimuli. The ring neuron RFs, however, are much coarser than those of simple cells, far larger and less evenly distributed across the visual field and respond mainly to orientations near the vertical. This suggests that ring neurons might have a less general function than simple cells [25]. In mammals, the very large population of simple cells means that small, high-contrast boundaries of any orientation are detected at all points in the visual field. Thus the encoding provided by simple cells preserves visual information and acts as a ‘general purpose’ perceptual network that can feed into a large number of behaviours. In contrast, the coarseness of the ring neuron RFs, allied to the tight relationship between specific behaviours and specific subpopulations of ring neurons, suggests instead that these cells are providing economical visual information that is likely tuned for specific behaviours [25].

To investigate such issues, we use a synthetic approach whereby investigations, in simulation, of the information provided by these populations of neurons can be related to behavioural requirements, thus ‘closing the loop’ between brain and behaviour. We show how the population code is well-suited to the spontaneous bar orientation behaviours shown by flies. Similarly, we verify that our population of simulated ring neurons is able to explain the success and failure of the fly to discriminate pairs of patterns. Upon deeper analysis, we demonstrate that certain shape parameters—orientation, size and position—are implicit in the ring neurons’ outputs to a high accuracy, thus providing the information required for a suite of basic fly behaviours. This contrasts with the rather limited ability of ring neuron populations (and flies) to discriminate between abstract shapes, casting doubt on cognitive explanations of fly behaviour in pattern discrimination assays.

## Results

Here we analyse the task-specific information provided by visually responsive ring neurons by simulating their responses during well-known behavioural experiments. To do this we use data from Seelig and Jayaraman [6] who used calcium imaging to examine the RFs of ring neurons, whose cell bodies are in specific glomeruli in the lateral triangle. As the RFs of glomeruli are remarkably consistent across flies [6], we combine them by averaging across flies to reduce measurement error and obtain sets of ‘canonical’ RFs, which can be thought of as visual filters. The averaging process assumes a certain amount of underlying homogeneity for each glomerulus across flies, which we feel is justified given their similar forms; the advantage of this approach, over one in which we, say, take the RF from a single fly, is that it reduces the inevitable noise that will have been accrued in determining the RFs for individual flies. Additionally, small changes in the averaging process have little effect on the results [26]. This process (for details, see Materials and methods) gave us a set of 28 R2 and 14 R4d filters (14 and 7 on each side of the visual field, respectively). We treat these as simple linear filters, following [6], as we are not attempting here to model outputs at the neuronal level. To investigate the visual information that these cells encode, we calculate outputs for a given visual stimulus by convolving it with the averaged ring neuron filters. This gives a population code where the outputs of the set of filters is the encoded ‘representation’ of the current visual stimulus. We interrogate these encodings to understand the information they contain, focusing on the relationships to specific behaviours.

### Orientation towards bar stimuli

**Fig 1:**
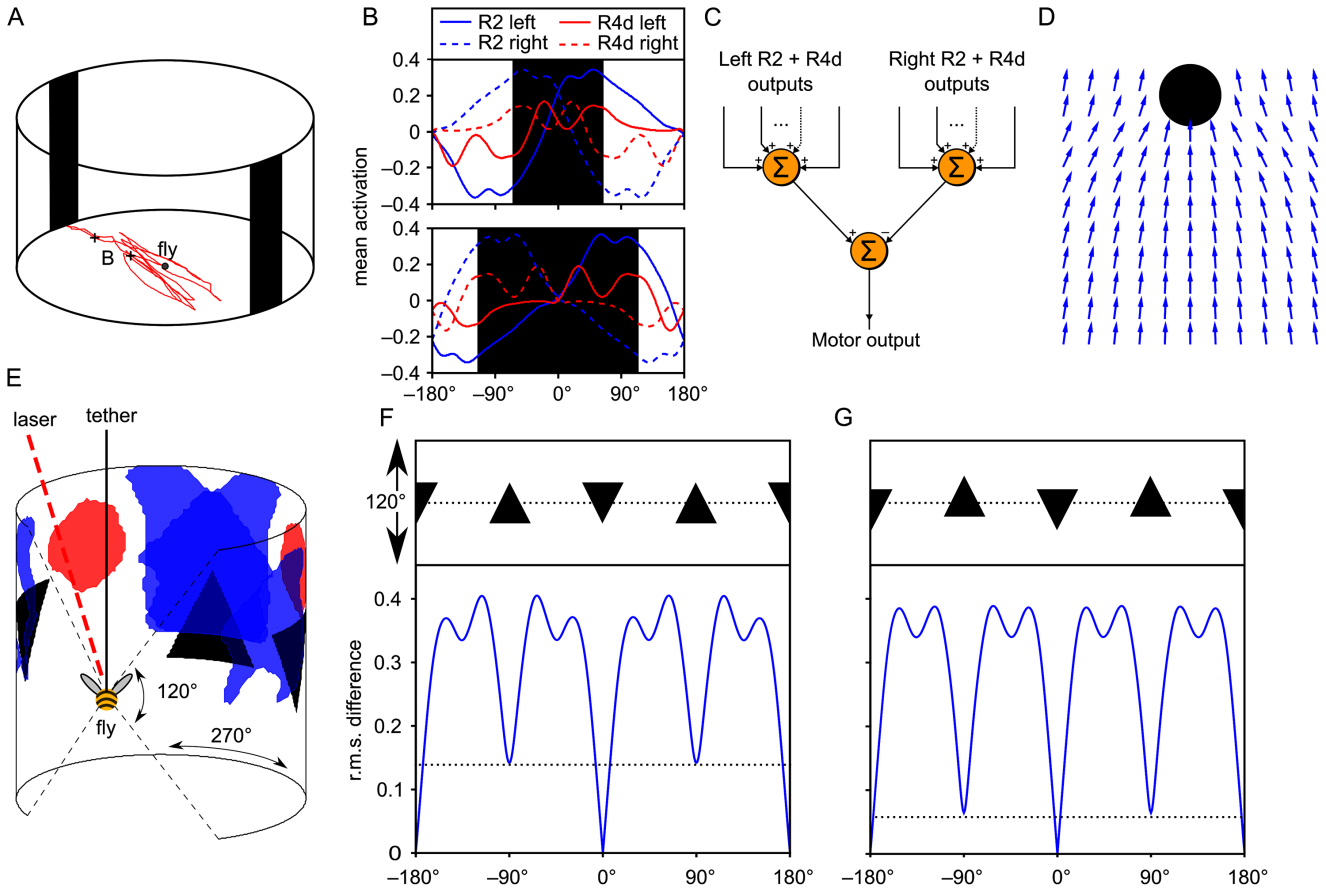
Simulation of two behavioural experiments with *Drosophila* to examine the outputs of the R2 and R4d RFs. A: A diagram showing Buridan’s paradigm [52, 53]. If a fly is placed in an arena between two large vertical bars, it will walk back and forth until exhaustion. B: The mean output of R2 (blue) and R4d (red) filters to bars of different widths from two points in the arena (indicated in panel A with crosses) across headings. C: The summed outputs of R2 and R4d filters can be used to drive orientation towards a bar stimulus with a simple proportional-integral-derivative (PID) controller. D: A vector field of orientations for a simulated fly driven by the simple PID controller. Note that this is not intended as a descriptive model of how bar attraction in flies operates, but as an illustration of the information latent in the outputs of the ring neurons, hence why many of the ‘flies’ in the vector plot would miss the bar. E: The fly is held tethered in a drum. As the fly attempts to rotate about its yaw-axis, the drum rotates in the opposite direction, thus allowing the fly to select the portion of the pattern in view. By monitoring the fly’s heading, one can surmise whether there is a spontaneous preference for one of the patterns. Whether the fly can learn to head towards one pattern is tested by adding a laser that punishes the fly for facing one of the patterns. Shown inside the drum are the visual RFs for one pair of left- and right-side glomeruli. F and G: The r.m.s. difference in output for R2 RFs as the pattern is rotated. The reference activities are the RF outputs when the simulated flies are at 0°. Patterns with a greater difference in activity at 0° *vs* 90° (indicated by dotted lines) should be more discriminable by flies. For two pairs of patterns we show that there is a much smaller difference in output when the triangles are aligned about the vertical centre of mass (G) than not (F). This mirrors real flies’ performance on this task [14].

We first consider experiments in which flies are presented with bar stimuli, as flies are known to spontaneously orient towards black bars [11], aiming for the centres of narrow bars and the edges of wide bars [27]. We therefore decided to examine the responses of simulated ring neurons to bars of different widths (Fig 1A and 1B). The summed outputs of the ensembles of ring neurons show peaks to the bars of different widths, which broadly matches experimental results (Fig 1B). For instance, R2 neurons respond maximally to the inside edges of large bars, while peak activity in R4d neurons occurs at bar centres and also at roughly ±90°. While we do not know the details of mechanisms downstream of the ring neurons and hence how their activity is transformed into action, the simulation is an existence proof that the information needed to control the observed behaviour is present in the sparse ring neuron code.

We further demonstrate this point by closing the loop between sensory systems and behaviour using a simple model of a fly viewing a bar in which the fly’s heading is controlled by the difference between the summed activation of left and right ring neurons (Fig 1C; see Materials and methods for details). The simulated fly approaches the bar from different distances, demonstrating centre-aiming when far from the bar and fixation of the edges when it is nearer and the bar’s apparent size is thus greater (Fig 1D). Through this example, we can see how the information present in this small population of visually responsive ring neurons can control a specific behaviour. We now turn to a more complex behaviour: pattern discrimination.

### Pattern discrimination in flies and ring neuron population codes

The standard paradigm for testing pattern discrimination involves putting a fly into a closed-loop system where it is tethered inside a drum, on the inside of which are two different visual patterns, alternating every 90°, giving four visual stimuli in total [7, 14, 15, 28] (see Fig 1E). As the fly attempts to rotate in one direction, the drum rotates in the other, giving the fly the illusion that it is moving in a stable world. To elicit conditioned behaviour, if the fly faces one of the four pattern stimuli it is punished by a heat beam. Over time, if the fly is able to differentiate the patterns, it should preferentially face the unpunished pattern. This procedure has been used to demonstrate that flies can differentiate stimulus pairs such as upright and inverted ‘T’ shapes, a small and a large square, and many others [14]. The ability to discriminate patterns in such an assay requires R2 neurons [7, 14, 29]. More specifically, synaptic plasticity afforded by *rutabaga* in these neurons is sufficient and necessary for observed pattern learning [15]. We therefore investigate the responses of ring neurons in simulations of the classic pattern discrimination paradigm.

To recreate the visual information perceived by flies in such experiments, we simulated the typical experimental flight arena with a fly tethered in the centre. We then examined the output of the ensembles of ring neurons for a fly rotating in the drum and looked at the difference in the activation code when the agent was facing the different patterns of a pair. Our logic is that if the ensemble codes were identical, it would be impossible for the patterns to be discriminated by interrogating the outputs of ring neurons alone. Similarly, the greater the difference in the ring neuron ensemble activation codes when looking at the pattern pairs, the easier they would be to discriminate (Fig 1F and 1G; see Materials and methods for details). Our discriminability measure is the root mean square (r.m.s.) difference between ensemble outputs when the (virtual) fly faces different azimuths in the drum. In this way, we can compare the ensemble output when the ‘fly’ is oriented at 0° (i.e. with the view centred on one pattern) and the ensemble output at other azimuths (Fig 1). We henceforth treat this as a measure of ‘discriminability’ of patterns, following the experimental work that we are modelling, though of course in reality an animal’s ability to discriminate stimuli is not an absolute value and varies depending on many factors, including task and training procedure [30]. The r.m.s. difference, as compared to the view at 0°, rises as the fly rotates in the drum, peaking as it faces the space in between the patterns and dropping to a minimum when facing the centre of the next pattern (Fig 1F and 1G). For some pairs of patterns, there is still an appreciable r.m.s. difference between the codes when facing the centres of each pattern, thus enabling their discrimination. However, in the example of Fig 1F and 1G, if we displace the patterns vertically, we see a drop in the r.m.s. difference between activation codes when the fly fixates the patterns. This is despite the fact that, to the human eye, the patterns still appear very different. Interestingly, the pattern pair in Fig 1G is also harder to discriminate for flies.

**Fig 2:**
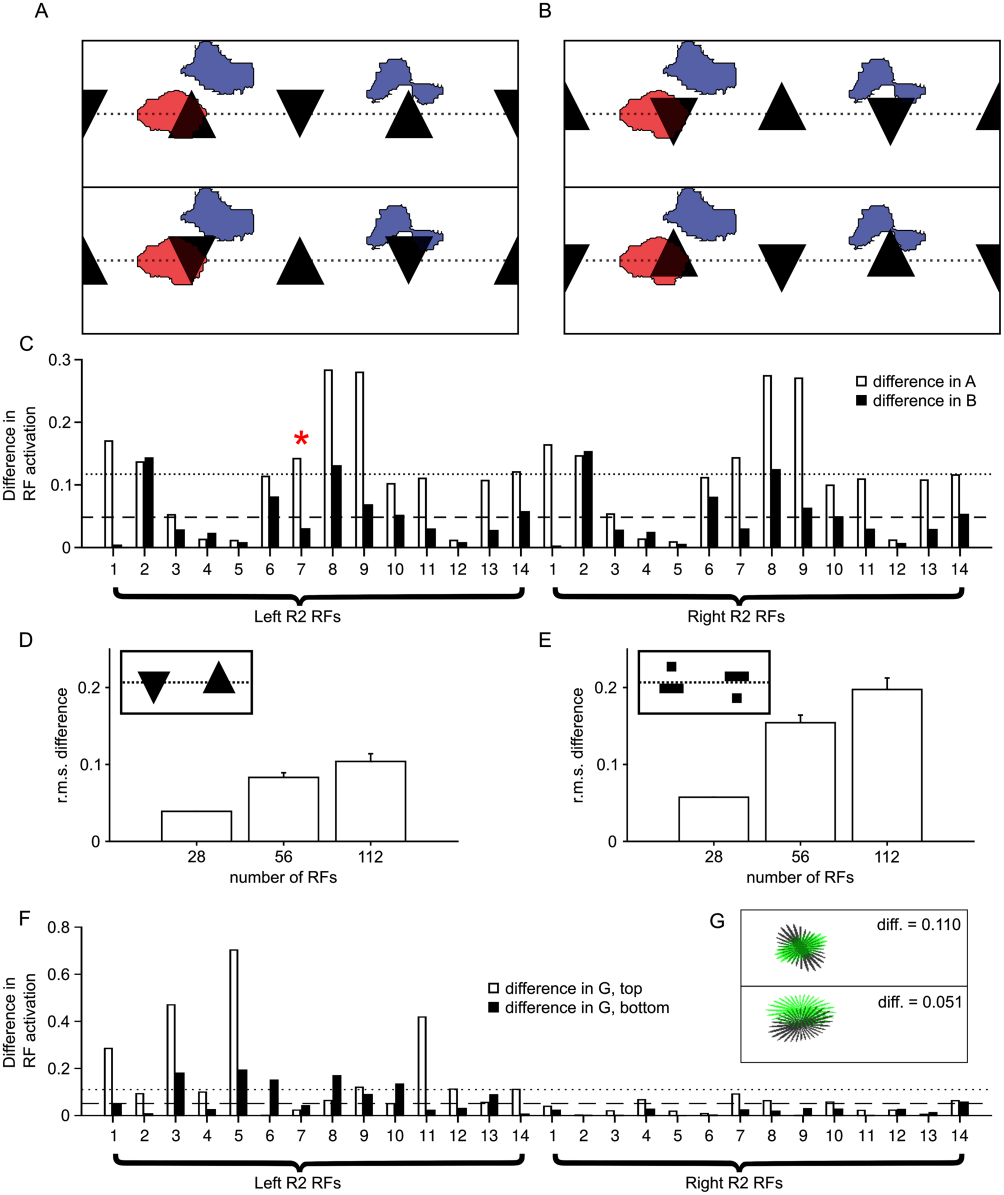
R2 cells do not encode detailed shape information. A–C: The discriminability of pattern pairs can vary greatly, independently of the apparent difference between visual stimuli. A: A pattern pair made up of an upright and an inverted triangle, aligned along their top and bottom. B: Another pattern pair, consisting of the same triangles as in A, but aligned about their vertical centres of mass. C: The difference in activation between 0° and 90° for all R2 RF filters for the triangles from A (white bars) and B (black bars). Discriminability is greater for the triangles in A than in B. The red asterisk marks the output of the ring neuron RF shown in A and B. D and E: Performance improves with number of RFs, for two sets of ‘difficult’ patterns (centre-of-mass-aligned triangles/bars, insets). Bars show mean r.m.s. difference between the activation of 28, 56 and 112 RFs to each pattern. For 28 RFs we use the R2 RFs. For 56 and 112 RFs, one and three extra sets of R2 filters, respectively, are added in random, non-overlapping positions on the visual field (see Materials and methods for details). As there is some stochasticity in this process, differences are averaged over 1000 trials with error bars showing standard deviation. F and G: We can also generate shapes that appear similar yet produce a large mean difference in RF activation (G, top) or appear different and produce similar RF activations (G, bottom). The stimuli here are ‘blobs’ of the form described in Materials and methods. To generate the stimuli, an optimisation was performed in MATLAB (fminsearch function) to minimise the ratio of blob difference to difference in activation (G, top) or its inverse (G, bottom). Pairs of stimuli are shown in grey and green whereas in the simulation both are black. F: The corresponding activations of the different R2 RFs to the blobs in G. Two similar patterns give a mean difference in activity of 11.0% and two very different patterns give a mean difference in activity of 5.10%. The dotted line indicates the mean activation for the blob in the upper panel and the dashed line for the blob in the lower.

**Fig 3:**
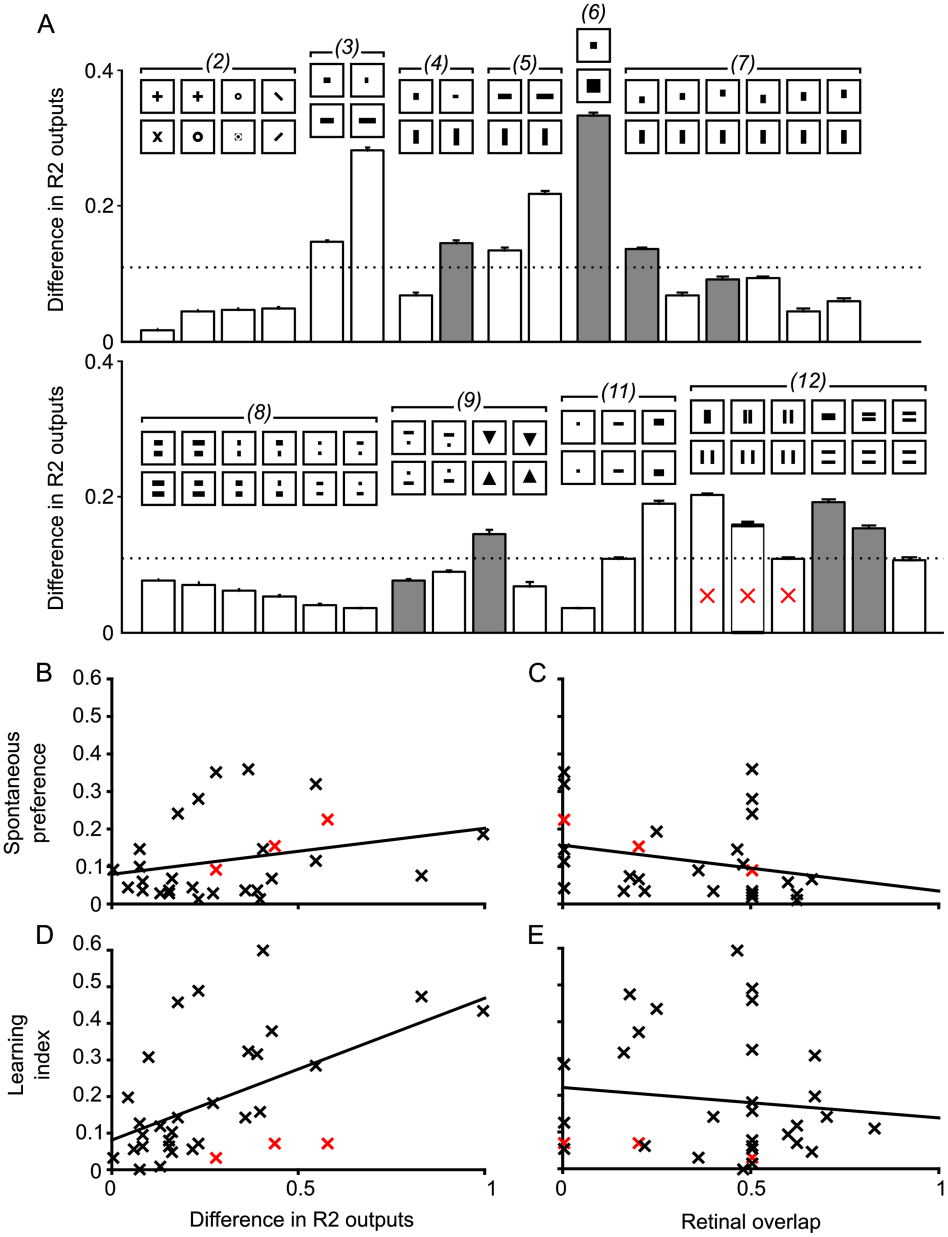
Outputs of simulated R2 cells and degree of retinal overlap for published pattern pairs. Difference in R2 RF output partly predicts whether patterns are discriminable by flies, but not whether there is a spontaneous preference. By contrast, retinal overlap predicts whether there is a spontaneous preference, but not whether the patterns will be discriminable. The patterns tested here are drawn from [14] and are grouped according to the figures in which they appear in that work; the corresponding figure numbers are shown in parentheses. All patterns for which the significance of ‘learning preference’ (‘$\bar{\text{DCP}}$’ in [14]) was given are included. A: Grey bars indicate that the learning index for the pattern was significant (i.e. *p* < .05 in [14]). A higher score indicates a greater r.m.s. difference in R2 activity and thus that the pattern was more discriminable by the simulation. Performance on more ‘horizontal’ patterns (e.g. *(3)* and the final three patterns in *(12)*) was poor in the behavioural experiments, but better in simulation. This is perhaps due to the horizontal motion of the patterns in training, as noted in [14]. B and C: Scatter plots of R2 difference (the ‘RF Model’) and retinal overlap (the ‘Retinotopic Model’) *vs* spontaneous preference (‘$\bar{\text{SCP}}$’ in [14]) shown in [14]. A significant correlation was found for the Retinotopic Model (*n* = 29; *ρ* = −0.370509; *p* < .05) but not the RF Model (Spearman’s rank, *n* = 29, *ρ* = .289, *p* = n.s.). D and E: Scatter plots of R2 difference (the ‘RF Model’) and retinal overlap *vs* learning index ($\bar{\text{DCP}}$). A significant correlation was found for the RF Model (Spearman’s rank, *n* = 32, *ρ* = .502, *p* < .005) but not the Retinotopic Model (*n* = 32, *ρ* = −.00960, *p* = n.s.). As two data points on the far right in panel D could be outliers, we reran the analysis excluding these points and found that the correlation was still significant, albeit less so (*n* = 30, *ρ* = .420, *p* < .05).

In this way, we can use the difference between ensemble codes when flies face the patterns to re-examine the discriminability of pattern pairs tested with flies. One illustrative example is shown in Fig 2 (see pattern set *(9)* in Fig 3), which contains pairs of ‘triangles’, one facing up and the other down. *Drosophila* are able to discriminate these pattern pairs when they are aligned along the top and bottom, but not when aligned about the vertical centres of mass [14]. Looking at the placement and form of the R2 RFs allows us to determine where this difference comes from (Fig 2). The excitatory regions of the RFs fall roughly across the middle of triangles that are not aligned about their vertical centres of mass and therefore the difference in width at this point will lead to differences in activation. If the triangles are offset (Fig 2B) so as to be aligned about their vertical centres of mass, their width will be similar for the regions of peak R2 coverage and the difference in activation will be lower. Thus the failure to discriminate features with an equivalent vertical centre of mass can be explained by the shape of the RFs interacting with the patterns directly. It is not necessary to invoke an additional system that extracts and compares the vertical centres of mass of the patterns.

Performance on poorly discriminated patterns can be improved, however, by simply adding more RFs of the same form. Fig 2D and 2E show the increase in performance with number of RFs for two such pattern pairs: triangles and triangularly shaped horizontal bars aligned about the centre of mass (from pattern set *(9)* in Fig 3; see Materials and methods for details). This demonstrates that the patterns could be discriminated by flies simply with the addition of more RFs centred on other portions of the visual field.

Similarly, pattern set *(2)* in Fig 3 gives examples of pattern pairs that are not discriminable by flies and also give only small differences in the outputs of R2 filters. This may seem surprising, given that these patterns appear quite different to human observers and are also very dissimilar if compared retinotopically. Thus we can see that the R2 ring neuron encoding is informationally sparse. Whilst the V1 region of human visual cortex contains neurons representing the full range of orientations across the visual field, R2 neurons have large RFs and poor orientation resolution. Hence, a pattern pair consisting of a diagonal line facing left and a diagonal line facing right, for example, have only a small difference in R2 outputs in our simulation and are also not discriminable by flies. This could, in the light of behavioural experiments alone, be interpreted as evidence that flies do not discriminate patterns on the basis of orientation. A more parsimonious explanation, however, is that the flies are failing because the form of the RFs means that the output code is similar for these particular orientations.

To emphasise the independence of apparent similarity of patterns and the visual encoding from R2 cells, we designed shape pairs that appear similar to humans, but are easily discriminable by the R2 population (white bars in Fig 2F), as well as shape pairs that are considered similar by the R2 population but not by human observers or in terms of retinal overlap (black bars in Fig 2F; see Materials and methods for details). Despite the similarity between the pairs of patterns, the first is readily discriminable, especially from the outputs of glomeruli 1, 3, 5 and 11, while the second pair—which we easily see as having a different orientation—has very low overall differences across the glomeruli. This shows that the irregular RF shapes can lead to counterintuitive results.

The small population of visually responsive R2 neurons can be thought of as a sensory bottleneck. If the information that passes through this bottleneck is all that a fly has available for pattern discrimination, then we should see a close relationship between the r.m.s. difference in simulated R2 output for a pattern pair and the flies’ ability to learn to discriminate that pair. We thus examined the difference in the outputs of the R2 filters between patterns from pairs drawn from work by Ernst and Heisenberg [14] (Fig 3). In general, the pattern pairs for which flies show a significant learned discrimination have a greater r.m.s. difference in R2 population activity [14]. All of the pattern pairs where flies show significant learning (*n* = 8) have R2 r.m.s. differences above the overall mean (Fig 3A and 3B), whereas 13 out of 18 patterns that flies found more difficult to learn had below-average r.m.s. differences (there were nine pattern pairs for which a significance level was not given that were excluded.) Across all pattern pairs, we find a significant correlation between the strength of the learning index reported for flies in [14] and the r.m.s. difference in R2 activation (Spearman’s rank, *n* = 30, *ρ* = .420, *p* < .05). Of course, these differences could simply result from the apparent similarity of the patterns. Therefore, as a control comparison, we quantified the similarity of pattern pairs based on the degree to which the patterns overlap in a pixel-by-pixel manner (see Materials and methods). There was no significant correlation with the flies’ learning index over the pattern pairs (Spearman’s rank, *n* = 32, *ρ* = −.068, *p* = n.s.). We additionally looked at the relationship between the two visual similarity metrics (R2 population code and pixelwise retinal overlap) and the degree to which flies show a spontaneous preference (i.e. without any conditioning) for one of the patterns within a pair (Fig 3D and 3E). There was no correlation for R2 population codes (Spearman’s rank, *n* = 29, *ρ* = .289, *p* = n.s.), but for retinal overlap there was a weakly significant correlation (Spearman’s rank, *n* = 29, *ρ* = −.371, *p* < .05). This is consistent with research showing that R2 neurons alone are critical for learned pattern differences [14], but not spontaneous preferences which, by contrast, seem to result from activity across all subsets of ring neurons [31].

There are, however, some discrepancies where the learning performance of flies for a particular pattern pair does not match the r.m.s. difference of our R2 population code. In some cases flies are better at discriminating pairs of patterns that differ along the vertical rather than horizontal axis (set *(3)* *vs* set *(4)*, and the pairs in set *(12)*, marked with red Xs in Fig 3). In contrast, the r.m.s. difference in the R2 population code discriminates horizontal and vertical patterns equally. This is because while our R2 filters are presented with static stimulus pairs to simulate a fly facing the centre of a pattern, for real flies the patterns were moving horizontally but fixed in the vertical axis making it harder for flies to resolve horizontal information [14].

Overall, we have shown that the behavioural performance of flies on a pattern discrimination task is approximated by a simple discriminability metric applied to the population activity of a small number of simulated R2 cells. There were, however, a number of seemingly ‘easy’ pattern pairs which neither flies nor the simulated population of R2 cells, perhaps surprisingly, could discriminate. On further investigation, we found that performance for poorly discriminated pattern pairs could be improved with the addition of extra R2-type RFs. Thus, it seems likely that the pattern discrimination capability of a set of R2-like neurons could easily have been improved over evolutionary time with the simple addition of more cells and we therefore suggest that there must have been little selection pressure specifically for a specialised pattern recognition module in fruit flies.

### What information is preserved in this simple neural code?

Information from 3000 ommatidia is funnelled to just 28 R2 and 14 R4d ring neurons, yet these cells are able to support a number of complex behaviours. We have shown how the R2 population code provides sufficient information to discriminate some pattern pairs, and also that, as performance could be improved with the addition of more ring neurons, general-purpose pattern recognition seems unlikely to be the purpose of the ring neuron system. So what information is this system tuned to extract? Examining the pattern pairs which flies and the R2 population were able to discriminate, we see that certain pattern parameters are implicitly coded for in the R2 population. Pattern sets *(6)* and *(9)* (Fig 3) suggest that, for instance, stimulus size and vertical centre of mass are parameters that can be recovered from the R2 population code after this sensory bottleneck.

**Fig 4:**
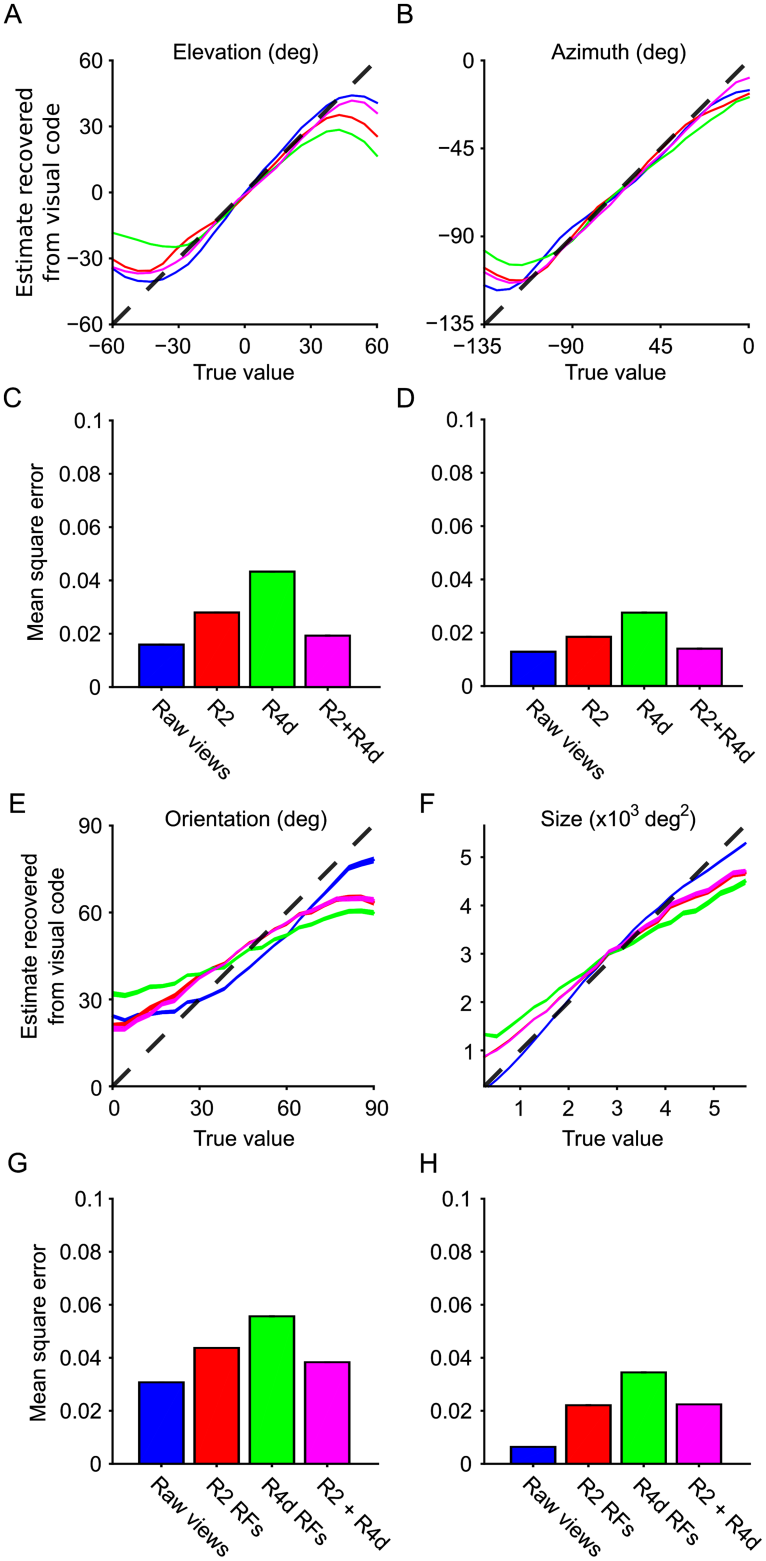
How much shape information is preserved in the R2 population code? ANNs were trained to estimate various properties of randomly generated ‘blob’ stimuli (*n* = 93,702) from raw views (3 × 14 = 42 pixels; blue), R2 neurons (*n* = 28; red), R4d neurons (*n* = 14; green) or R2 and R4d neurons (*n* = 42; magenta). Panels A–D show results for networks trained with elevation and azimuth and panels E–H for orientation and size. For each visual input a network was trained with 100 training cycles and average performance with blobs that were not part of the training set was taken. A and B: Plots of elevation and azimuth of the test visual stimuli *vs* the mean network output (*n* = 140,554). The dashed line indicates ideal performance (i.e. *y* = *x*) and the thickness of the lines at each point shows standard error. The possible values of elevation and azimuth were constrained by the size of the fruit fly visual field (approx. 120° × 270°). Within this range there were 22 possible values. C and D: Average network performance (r.m.s. error) for networks trained to recover elevation (C) or azimuth (D) for each type of visual input (colour code as above; *n*(train) = 4259, *n*(test) = 6389). Standard error is shown, but is very small. E and F: Network performance in recovering stimulus orientation and size. Orientation was constrained between 0° and 90°, to avoid the problem of aliasing, and varied with 22 levels. G and H: Average network performance (r.m.s. error) for networks trained to recover orientation (G) or size (H) and for each type of visual input (colour code as previously). Standard error is shown, but is very small.

We now address in more general terms the question of what shape information is implicitly conveyed in the ring neuron population code. To do this, we generated large sets of ellipse-like ‘blob’ stimuli varying in size (specified by major-axis length), position (azimuth and elevation) and orientation. The blob generation procedure was stochastic and so the precise shape of each blob was random and unique (see Materials and methods). We then trained an artificial neural network (ANN) to recover this shape information from either a raw image of the shape (the control condition) or from the output of the R2/R4d populations on presentation of the blobs. We are using ANNs here as statistical engines interrogating the output of the ring neuron population code to determine if shape information is implicit to the code and has therefore passed through the sensory bottleneck. We first examined whether ANNs could be trained to extract positional information (the elevation and azimuth) of randomly generated blobs. Note that as the blobs are initially aligned about their centres of mass, elevation is equivalent to vertical centre of mass, except where the blobs are partially outside the visual field. The blobs varied along four parameters: elevation (≥ −60° and ≤ 60°), azimuth (≥ −135° and ≤ 0°), orientation (≥ 0° and ≤ 90°) and major-axis length (≥ 12.79° and ≤ 60°). Each parameter had 22 possible values, giving a total of 234,256 (= 22^4^) stimuli. Of these, approximately 40% (*n* = 93,702) were used for training and the remainder (*n* = 140,554) for testing. Results for the test set (Fig 4A–4D) show that ANNs are indeed able to extract information about elevation and azimuth from any of the input types (‘raw view’, ‘R2’, ‘R4d’ or ‘R2 + R4d’). Performance was better with parameter values near the middle: at the extremes, portions of the stimuli lay outside the visual field of the simulated fly, meaning stimuli begin to disappear ‘off the edge’ of the visual field (Fig 4A and 4B), making the task harder (i.e., is this a large object projecting outside the visual field, or a smaller object at the edge?). While performance was best with raw views as inputs (Fig 4C and 4D), positional information could still be reliably extracted from ring neuron outputs. The R2 code performs better than the R4d and the addition of R4d RFs to the R2 code (‘R2 + R4d’), while adding dimensionality, does not improve performance, suggesting that either an R2-like encoding is sufficient to extract positional information, or that the information in the two codes is redundant. Thus small populations of ring neurons retain positional information.

We next trained ANNs to decode information about stimulus orientation and size. The stimuli were random blobs, as before, with the same possible values for elevation, orientation and size. This time, however, azimuthal position was fixed at −90°. The reason for this was that the neural network struggled to encode information about orientation when azimuth also varied, presumably because the centres of the receptive fields—and thus the position on the visual field where they can best extract information—are clustered at around −90°. For this experiment there were therefore 10,648 (= 22^3^) stimuli, of which approximately 40% (*n* = 4259) were used for training and the remainder (*n* = 6389) for testing. The ANNs were able to extract this shape information from both raw images and the ring neuron outputs (Fig 4E–4H). Orientation was the parameter with the highest error score, possibly because its calculation requires a second-order statistic (the covariance of the shape). Nonetheless, both parameters could be simultaneously estimated by an ANN neural network fed with ring neuron outputs.

In summary, we have shown that information about a number of shape properties passes through the bottleneck created by the small number of ring neurons. This indicates that such information is available downstream of the ring neurons for the guidance of behaviour.

## Discussion

A general problem in neuroscience is understanding how sensory systems organise information to be at the service of behaviour. Computational approaches can be important in this endeavour, as they allow simulation of the sensory experience of a behaving animal, such that one can investigate how this information is transformed by populations of neurons. In this way, we can relate the details of neural circuitry to theories about the requirements of behaviour. Work by Seelig and Jayaraman [6], showing the forms of visual receptive fields for ring neurons projecting to the ellipsoid body of flies, gave us the opportunity to investigate how these neurons transform information and how this information relates to specific behaviours. In particular, we have shown that despite the small size of the neural code, the outputs of the ring neurons can subserve both bar following and limited pattern recognition and also implicitly convey information about shape parameters. We now discuss the implications of our findings.

### Short-term memory for object position in flies

One striking feature of the ring neuron receptive fields is that they are in general tuned to vertically oriented objects. We know that fruit flies are strongly attracted to vertical bars, a finding that has been leveraged across a range of behavioural paradigms (e.g. bar fixation: [8]). In one, individual flies are placed into a virtual-reality arena with two vertical stripes 180° apart: flies will typically head back and forth between the two bars repeatedly. Occasionally, when a fly crosses the arena’s midline, the bars disappear and a new bar is presented at 90° to the originals, to which the flies reorient. The new target then also disappears, and the flies resume their initial heading, even though the original bar is no longer visible. This indicates that directional information is stored in short-term memory and updated. Work by Neuser and colleagues [8] has shown that R4 (and R3) ring neurons are involved in this spatial orientation memory. We found that both R2 and R4d neurons were responsive to vertical bars of varying widths, mimicking flies’ preference for the edges of larger bars and the centres of narrower ones [27]. We also showed that the cells provide sufficient information to guide homing towards a large vertical object and, separately, that the azimuth of bar stimuli makes it through the sensory bottleneck. Taken together, these findings demonstrate a viable role for the small R4d population in the behaviours described above.

The more general role of R4d cells within the central complex is still unknown. There is evidence that R4d neurons are able to act as a ring attractor, maintaining a stable encoding of the fly’s orientation with respect to a landmark [9, 32]. Therefore, R4d neurons could be conceived variously as functioning like mammalian head-direction cells [33], playing a part in a path integration system [8] or in conditioning of visual orientation [34]. These possibilities are not mutually exclusive, of course, and their true function (or functions) will become apparent only with a better understanding of the behaviours in which they are involved.

### Do flies recognise patterns?

*Drosophila* can discriminate patterns differing in size, orientation and elevation and other complex shape parameters, an ability for which R2 cells are critical [7, 14, 15]. We have shown that the discriminability of a given pattern pair is predicted by the outputs of the small population of R2 cells, which have coarse receptive fields and therefore do not encode higher-order visual properties explicitly. Does this limited ability of the R2 population (and, of course, the fly) to discriminate patterns suggest that flies might be a good model for the study of a universal perceptual process of pattern recognition, or might limited pattern recognition be an artefact of a perceptual system tuned to other tasks? Any selection pressure on flies’ ability to discriminate patterns (as bees need to do, for instance) would surely have led to a larger R2 population or, possibly, visual input to the mushroom body [35, 36], and we can therefore be confident that ring neurons have not been tuned for arbitrary, general-purpose pattern recognition. Accordingly, we must suggest caution if research on flies is used with the aim of understanding the neural basis of pattern recognition or even visual cognition more generally [37]. So what behaviours are served by the information that makes it through this sensory bottleneck?

It is interesting to consider to what extent *Drosophila*’s ecological needs are served by general learning mechanisms—such as a capacity to learn arbitrary visual stimuli—and to what extent by domain-specific abilities. For example, bees have a well-attested ability to learn many varied patterns, which presumably derives from a need to learn about flowers [38]; it is not apparent, however, that there has been a comparable selection pressure on *Drosophila* for such general-purpose learning. Across the animal kingdom there are many cases where a task-specific heuristic can provide an elegant solution. For example, male fiddler crabs (*Uca pugilator*) treat salient objects above the horizon as predators and everything below as conspecifics [39]. Similarly, *Drosophila* have a mechanism to approach bars and to avoid small objects [13]; presumably to approach vegetation (for oviposition, etc.) and avoid predators, respectively. In order to fully understand these circuits we need to examine further how flies depend on a balance of innate visual responses versus learned visual information.

So, if the R2s are not truly ‘pattern-recognition cells’, the question remains: what are they for? Though we have not attempted to answer this question here, we have shown that there is *implicit* information about higher-order properties, such as stimulus position, orientation and size, in the RFs’ code, which could drive any number of natural behaviours. For example, elsewhere we have shown that the information content of ring neuron RFs is suitable for place learning and homing [26], and although this behaviour in flies involves a subset of ring neurons other than those examined here (R1), it gives an indication of how small populations of coarse, wide-field cells can be used to drive behaviour.

### Summary

The goal of this work was to investigate the information encoded in a population of visually responsive ring neurons, in simulations of classic pattern discrimination assays. Our aim was to examine the behavioural uses to which the information encoded in this population of cells could be put by a fruit fly. Of course, a full understanding of these neurons requires detailed knowledge of how they interact with other neural circuits for behaving flies in natural environments. Hence future work needs to address the interaction between brain, behaviour and environment [40].

For the brain, a sensible starting point is to ask how ring neurons and the information they carry are integrated in the central complex circuitry. Recent work has shown the presence of a ring attractor network [9, 32, 41, 42] in the ellipsoid body of the central complex which integrates both visual and proprioceptive information. This circuit is able to retain a heading in short-term memory [8] and thus the cells we have modelled could be useful in contributing information about the position of behaviourally relevant objects. Of course, there are many details to be determined, such as the dynamics of neural coding in this circuit and the sensory pathways that lead to the observed receptive fields [43]. In the current study we have not considered neural dynamics and have assumed that the information would be extracted as rate codes. While this is a common assumption for models of visual perception (e.g. [24]) we note that information could be extracted via a timing code, perhaps even more efficiently—especially if the fly is actively perceiving its environment. Though it is possible to convert from an analogue neural network to a spiking neural network [44], more work would be needed to establish this. Finally, the story is complicated further by the sheer variety of behaviours in which these cells have been implicated: for example, different subsets of R2 neurons have also been implicated in an olfactory decision task [45] and in sleep drive [46].

**Fig 5:**
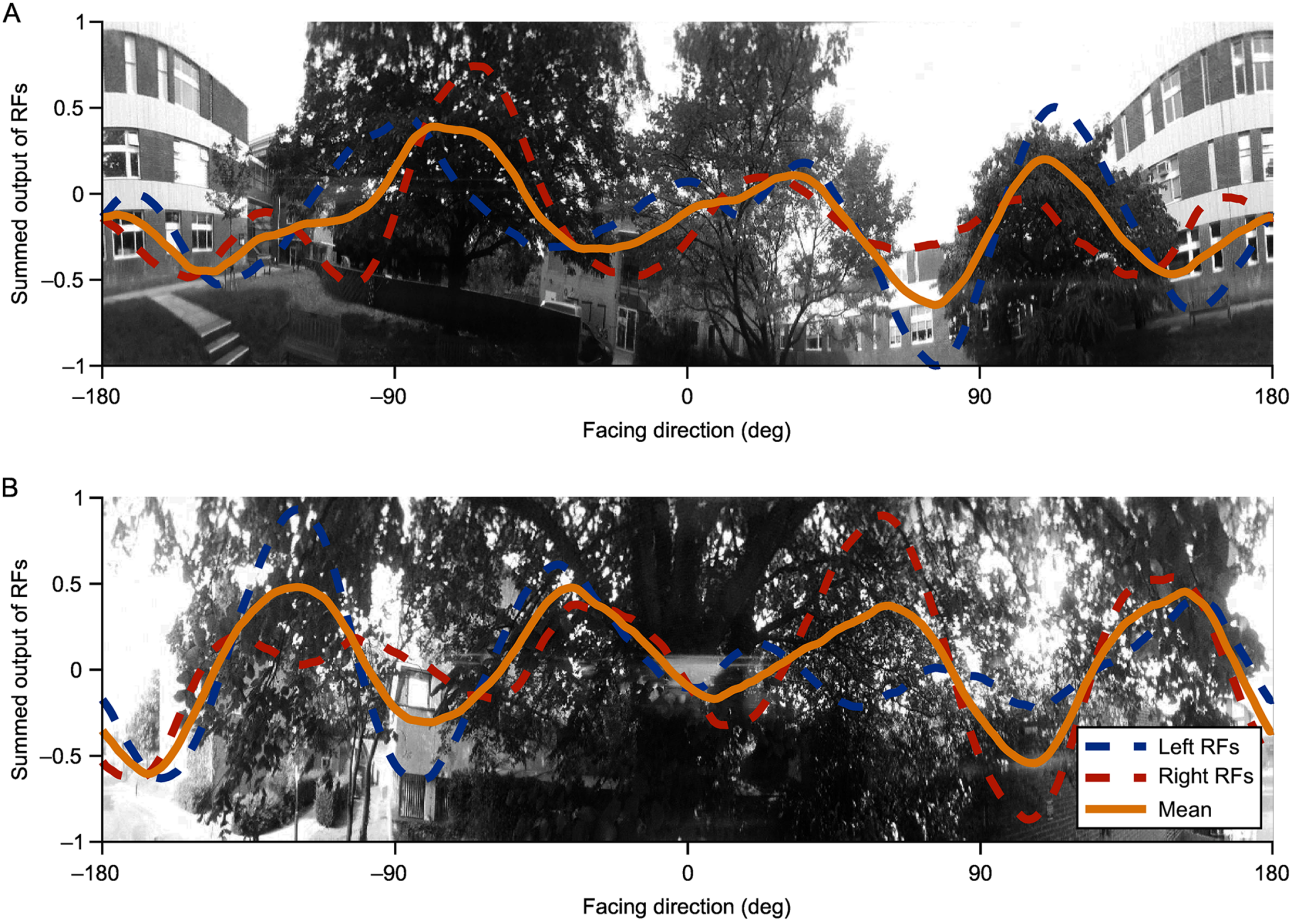
Mean output of R4d RFs as they move azimuthally across two natural panoramic images. Mean across all R4d RFs is shown in yellow together with the left- and right-side RFs (blue and red dashed lines, respectively) with values normalised to the greatest absolute mean value of left or right RFs across all azimuths and both images (i.e. the *y*-scale is the same for A and B). A: Response of filters to distant trees. Many R4d RFs respond preferentially to the positions of trees, meaning outputs—both mean, left and right—peak in the centre of trees and tend to dip when facing azimuths with more sky or lighter areas. There are minor differences between the direction at which left and right signals peak, but there is sufficient information in the mean responses to allow a fly to head towards large vertical objects from a distance. B: Response of filters to a nearby tree. Mean responses again peak when facing salient, vertically oriented portions of the image (hanging branches) and dip at lighter portions, meaning flies could identify and head towards these regions. While left and right responses peak together in some cases, for the peaks at –120° and 60° there is an asymmetry in the left and right signals which could provide steering information to allow a fly to navigate towards these targets in a similar fashion to the model used in Fig 1A–1D.

The sensory ecology of fruit flies is still largely a mystery (see [47]), despite the immense promise and productivity of *Drosophila* neuroscience research. We thus know relatively little about ‘natural’ *Drosophila* visual behaviours—in contrast to bees, for which we know much about behaviour but comparatively little about the nervous system. One pertinent example of this is visual pattern recognition, where for bees we have a good understanding of the real-world challenge facing a forager. This has enabled models of pattern recognition to be developed for bees [48]. Without a detailed understanding of sensory ecology and the natural behaviour of flies, it is hard to understand what type of pattern vision flies might need. However, some fly behaviours are easier to relate to the natural environment. Flies show an innate attraction to long bar-like objects, on which they might perch [13] and in Fig 5 we show one example of how behaviourally relevant information is maintained in the output of R4d cells even for complex scenes. Taking this further, we could consider how an active fly in a complex environment might be able to shape its own visual input to give us a better understanding of the true potential of the fly as a pattern discriminator. More generally, it is only with a deeper knowledge of fruit fly ecology that we will be able to close the loop between brain, body and environment and thus obtain a full understanding of the whole system.

One of the advantages of studying insects is the potential for describing their neural processes with modelling. In this way, simulations can help bridge the gap between biology and behaviour [49]. We have shown that the sensory bottleneck produced by small populations of cells is not a barrier to the specific information that is required for particular behaviours. However, this modelling work, and the neuroscience that invited it, do suggest caution when proposing that flies possess general-purpose visual cognition. We thus hope that future experiments, grounded in both the ecological needs of the animal and the information given by neural circuits, will be able to better inform the next generation of models, and vice versa.

## Materials and methods

### Neurogenetic methods used for estimating ring neuron receptive fields

The goal of Seelig and Jayaraman’s work [6] was to examine responses of lateral triangle microglomeruli (which house the cell bodies of the ring neurons) to visual stimuli. For this, they employed two-photon calcium imaging to examine the activity of genetically targeted subsets of microglomeruli, the R2 and R3/R4d neurons. Fluorescence was recorded for head-fixed flies held in an arena with a curved display composed of an LED array. In order to map the RFs, the flies were presented with a series of flashing dots at random locations on the visual display; the fine structure of the RFs was then revealed by using white-noise stimuli [50]. The accuracy of the estimated receptive fields was then verified by correlating predicted with actual responses to novel bar stimuli (and a high degree of correspondence was found). The predicted responses were calculated by using the RFs as linear filters through convolution and so we follow a similar procedure here.

### Turning visual receptive field data into visual filters

**Fig 6:**
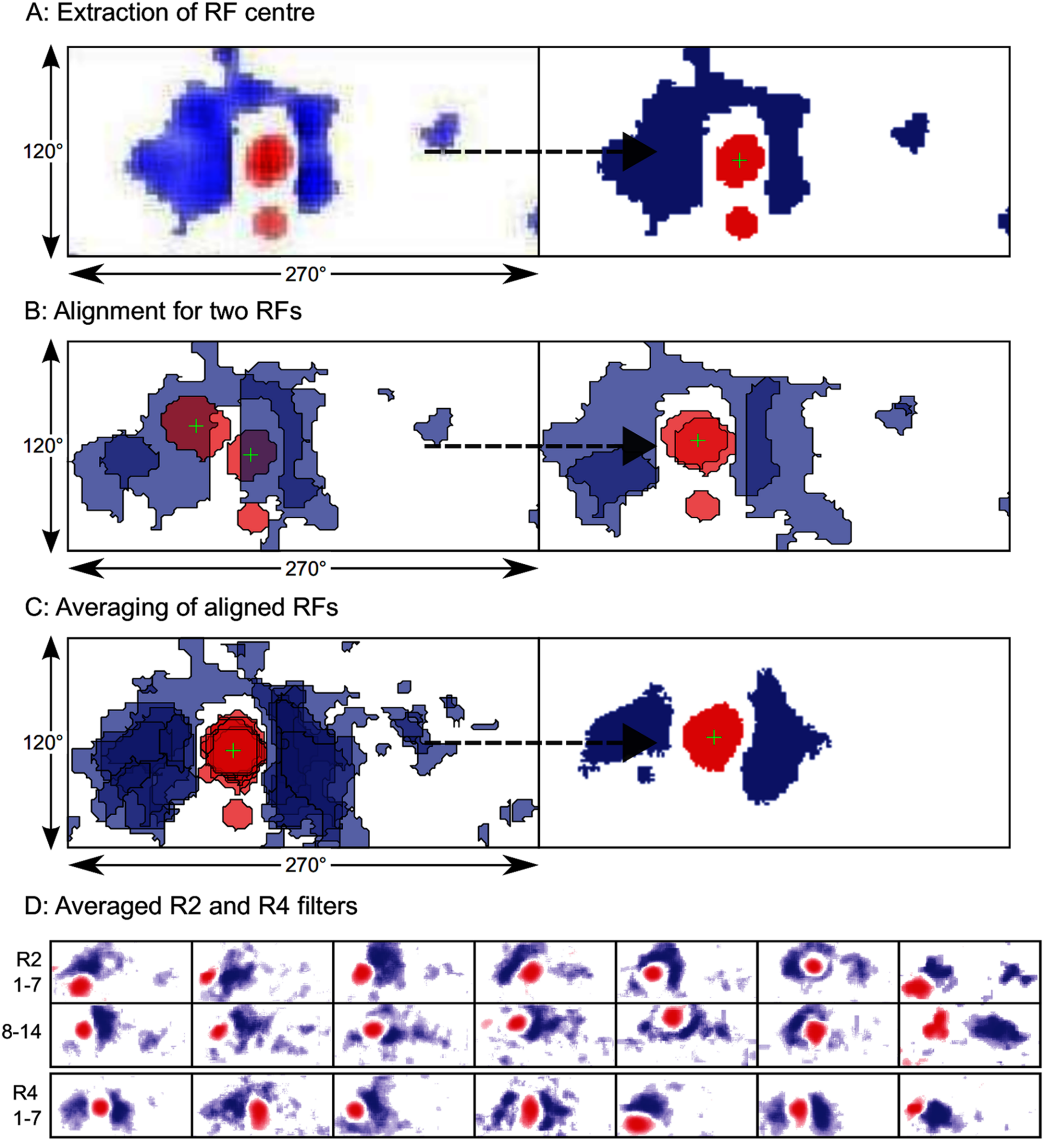
The algorithm for obtaining average RFs. A: The raw image (left; fly glomerulus 1) is thresholded so as to give excitatory and inhibitory regions of uniform intensity (right). The ‘centre’ is then calculated as the centroid of the largest excitatory region (+). B: Aligning two RFs. The new centre is taken as the average of the centre of both RFs and the RFs are then shifted so that the centres are aligned. C: Averaging the RFs for this glomerulus over all flies (*n* = 7), following alignment. Note that this is the left-side version; the right-side version is its mirror. D: Averaged R2 and R4d filters.

To create the visual filters which represent the RFs, we first extract the image representations of the RFs from Seelig and Jayaraman (Extended Data Figure 8 in [6]). This gives us images of 112 × 252 pixels for R2 neurons and 88 × 198 pixels for R4d. Given the visual field is taken as 120° × 270°, this corresponds to a resolution of 1.07° and 1.36° per pixel, respectively. As data is given for multiple flies, we averaged the RFs for the different glomeruli across flies (2 ≤ *N*(*R*2) ≤ 6, 4 ≤ *N*(*R*4) ≤ 7). This process is summarised in Fig 6. Each pixel on the extracted image is initially assigned a value ranging from –1 for maximum inhibition to 1 for maximum excitation, based on the values given by the colour scale bars in [6]. These images are then thresholded to give a kernel *g*(*i*, *j*):

$$\begin{array}{c} g\left(i,j\right)=\{\begin{array}{cc} 1 & \text{for}\;R_{i,j}\geq T;\\ -1 & \text{for}\;R_{i,j}\leq -T;\\ 0 & \text{otherwise.} \end{array} \end{array}$$

where *g*(*i*, *j*) is the (*i*, *j*)th pixel of the kernel, *R*_*i*,*j*_ is the (*i*, *j*)th value of the processed RF image and *T* is the threshold value, here 0.25 (Fig 6A).

We take the centroid of the largest excitatory region as the ‘centre’ of each of the kernels. The excitatory region is then extracted using MATLAB’s bwlabeln function (with eight-connectivity) and its centroid, (*x*, *y*), with the regionprops function. The mean centroid, $\left(\bar{x},\bar{y}\right)$, across flies is then calculated and the kernels are all recentred on this point:

$$\begin{array}{c} \hat{g}\left(i,j\right)=\{\begin{array}{cc} g(i+y-\bar{y},j+x-\bar{x}) & \text{for}\;1\leq i+y-\bar{y}\leq m\;\text{and}\;1\leq j+x-\bar{x}\leq n;\\ 0 & \text{otherwise.} \end{array} \end{array}$$

where $\hat{g}\left(i,j\right)$ is a recentred kernel (Fig 6C).

In order to calculate the activation for a given RF on presentation of an image the RF must first be resized to have the same number of pixels as the image. This is accomplished by resizing the average RF, $\bar{g}\left(i,j\right)$, using Matlab’s imresize function with bilinear interpolation and then scaled to [−1, 1]. Finally, the filter is thresholded and the excitatory and inhibitory regions are assigned different normalised values:

$$\begin{array}{c} K_{i,j}=\{\begin{array}{cc} \bar{g}\left(i,j\right)÷S_{\text{exc}}, & \text{for}\;\bar{g}\left(i,j\right)>0;\\ -\bar{g}\left(i,j\right)÷S_{\text{inh}}, & \text{for}\;\bar{g}\left(i,j\right)<0;\\ 0, & \text{otherwise.} \end{array} \end{array}$$

where *S*_exc_ and *S*_inh_ indicate the sums of excitatory and inhibitory pixels, respectively. This method of normalising values has the result that the activation (see below) for an all-white or -black image will be zero. Other normalisation schemes are possible, but the choice is somewhat arbitrary, as we are only interested in the differences in output values. Furthermore, RFs are sensitive to contrast differences, so a zero-sum filter, as seen in edge detectors, is appropriate. Additionally, assigning biologically relevant values is not possible because of a lack of data.

The activation of an average kernel, *K*, to the presentation of a greyscale image, *I*, at rotation *θ*, is then:

$$\begin{array}{c} \begin{array}{cc} A\left(I,K,\theta \right)=\sum_{i=1}^{m}\sum_{j=1}^{n}I_{i,j}\left(\theta \right)K_{i,j}, & \text{where}\;0\leq I_{i,j}\left(\theta \right)\leq 1 \end{array} \end{array}$$ (1)

where *I*_*i*,*j*_(*θ*) and *K*_*i*,*j*_ are the (*i*,*j*)th pixels of the image and kernel, respectively.

### Replication of behavioural experiments

The equation for describing the bar fixation mechanism shown in Fig 1C is as follows:

$$\begin{array}{c} \phi_{\text{turn}}=G\cdot \frac{\pi }{4}(\sum_{K\in G_{\text{left}}}\max\left(0,A\left(I,K,0{}^{\circ}\right)\right)-\sum_{K\in G_{\text{right}}}\max\left(0,A\left(I,K,0{}^{\circ}\right)\right)) \end{array}$$

where *I* is the view of the bar from the agent’s current location and **G**_left_ and **G**_right_ are the sets of left- and right-side filters. ‘*G*’ is a parameter to control the gain of the system, and here was set to 2.

For the pattern recognition tasks (see Fig 3), the difference in activation is calculated as follows:

$$\begin{array}{c} D\left(I\right)=\sqrt{\frac{\sum_{K\in G}{\left(A\left(I,K,0{}^{\circ}\right)-A\left(I,K,90{}^{\circ}\right)\right)}^{2}}{\left|G\right|}} \end{array}$$

where **G** is the set of R2 filters, *I* is the current pattern pair and *A*(⋅, ⋅, ⋅) is the activation of the kernel to the pattern, as described in Eq 1. The choice of r.m.s. difference as a difference function is somewhat arbitrary, but r.m.s. difference is commonly used (e.g. [51]); alternatively one could use mean absolute difference or mutual information etc., although the choice is not critical as we are looking at relative differences and the RF output code is normalised.

The retinal overlap for two binary patterns, *A* and *B*, is calculated in two steps. Firstly we measure the number of overlapping pixels between *A* and *B*; this value is referred to as *Q*. Next the proportion of pixels which this overlap represents for *A* and *B* is calculated and, finally, we calculate the retinal overlap as the average of these two values:

$$\begin{array}{c} O\left(A,B\right)=\frac{Q}{2}(\frac{1}{\sum_{i=1}^{m}\sum_{j=1}^{n}A_{i,j}}+\frac{1}{\sum_{i=1}^{m}\sum_{j=1}^{n}B_{i,j}}) \end{array}$$

where *A*_*i*,*j*_ and *B*_*i*,*j*_ represent the *i*th, *j*th pixel of patterns *A* and *B*, respectively.

We also carried out simulations of pattern discrimination where the number of R2 RFs was varied between 28, 56 and 112 (Fig 2D and 2E). The ‘28-kernels’ condition simply used the original kernels in their original positions (see Fig 6D). For the 56- and 112-kernel conditions (double and quadruple the number of kernels, respectively), the original kernel types and positions were used for the first 28 kernels for every trial and the remainder were placed in random locations, with the only constraint being that the ‘centre’ of the new kernel could not be within 10° of the centre of any already-placed kernel. Equal numbers of each kernel type (corresponding to each one of the original R2 filters) were used in each condition; that is, each kernel type was repeated twice for the 56-kernel condition (once in the original position and once in a random position) and four times for the 112-kernel condition (once in the original position and three times in a random position). The kernels were also shrunk for the latter two conditions by a factor of $\frac{1}{\sqrt{2}}$ and $\frac{1}{2}$, respectively, to keep the sum of the retinal area covered by all kernels constant across conditions, although the shapes of the kernels were otherwise kept the same. As the kernels were in fixed positions for the first condition, only one test was performed; for the other two conditions, 1000 trials were carried out.

### Neural networks

The ANNs were implemented using the Netlab toolbox for MATLAB. All networks were two-layer feedforward networks, with 10 hidden units and a linear activation function for the output units. There were 100 training cycles and optimisation was performed with the scaled conjugate gradient method.

#### Random blob stimuli

The stimuli used to train the networks were a series of black ‘blobs’ on a white background. The blobs were based on ellipses with a fixed ratio between the lengths of the major and minor axes (2 : 1), with the radii modified with complex waves:

$$\begin{array}{c} r\left(\theta \right)\leq {\left(\frac{{\text{cos}}^{2}\theta }{2}+\frac{{\text{sin}}^{2}\theta }{a}\right)}^{-1}+W\left(\theta \right),\theta \in \left\{0,2\pi \right\} \end{array}$$

where *a* is the length of the major axis and *W*(*θ*) is a complex wave defined as:

$$\begin{array}{c} W\left(\theta \right)=\sum_{i=1}^{n}W_{i}\left(\theta \right)=\sum_{i=1}^{n}A_{i}\sin\;f_{i}\left(\theta +\phi_{i}\right) \end{array}$$

where *A*_*i*_, *f*_*i*_ and *ϕ*_*i*_ describe the maximum amplitude, frequency and phase shift of the wave, *W*_*i*_(*θ*), respectively. This method for generating stimuli allows for a substantial degree of random variation between the stimuli, while not producing shapes that are so irregular as to be unlearnable by a neural network. In these experiments, *A*_*i*_, *f*_*i*_ and *ϕ*_*i*_ were randomly generated and *n* = 2. *A*_*i*_ was a random value from 0 to 1, *f*_*i*_ were random integers from 1 to 30 and *ϕ*_*i*_ was a random value from 0 to 2*π*.

The blobs were first generated, according to the above equation, as images of 120 × 270 pixels. For the ‘raw view’ stimuli, these images were resized, using MATLAB’s imresize function, to 3 × 14 pixels, thus giving the same number of inputs as there are R2 and R4d filters (*n* = 42).
